# Retraction Note: Performance of sustainable self-compacting fiber reinforced concrete with substitution of marble waste (MW) and coconut fibers (CFs)

**DOI:** 10.1038/s41598-023-39394-x

**Published:** 2023-08-01

**Authors:** Jawad Ahmad, Fahid Aslam, Rebeca Martinez-Garcia, Mohamed Hechmi El Ouni, Khalid Mohamed Khedher

**Affiliations:** 1 Department of Civil Engineering, Military College of Engineering, National University of Sciences and Technology, Risalpur, 24080 Islamabad Pakistan; 2 Department of Civil Engineering, Prince Sattam Bin Abdulaziz University, Al-Kharj, 11942 Saudi Arabia; 3 Department of Mining Technology, Topography and Structures, Campus de Vegazana S/N, University of León, 24071 León, Spain; 4 Department of Civil Engineering, College of Engineering, King Khalid University, 61421 Abha, Saudi Arabia; 5 Applied Mechanics and Systems Research Laboratory, Tunisia Polytechnic School - University of Carthage, La Marsa, 2078 Tunis, Tunisia; 6 Department of Civil Engineering, High Institute of Technological Studies, Mrezgua University Campus, 8000 Nabeul, Tunisia

Retraction of: *Scientific Reports* 10.1038/s41598-021-01931-x, published online 30 November 2021

The Editors have retracted this article.

After publication concerns were raised that the XRD spectra in Fig. 8 are identical. The authors are unable to provide the original data for examination. In addition, an investigation by the Editors has shown inappropriate changes in authorship during the review process. The Editors no longer have confidence in the results and conclusions presented.

Jawad Ahmad disagrees with this retraction. Fahid Aslam and Mohamed Hechmi El Ouni did not respond to correspondence from the Editors about this retraction. The Editors were not able to obtain current email addresses for Rebeca Martinez-Garcia and Khalid Mohamed Khedher.

